# 

**Published:** 1920-05-15

**Authors:** 


### THE COLLEGE OF NURSING.

**(Limited by Guarantee.)**

### FIFTH ANNUAL REPORT OF THE COUNCIL.

Every year the Council, in presenting its Annual Report to Members of the College, has struck a hopeful note, but never with more confident assurance than this year, when it submits its Fifth Annual Report. The College during the last four years has grown so rapidly that apprehension might have been felt lest the growth were too fast for its strength. There is now, however, a feeling of solidarity within the College, which is the outcome of increased interest and activity from individual members, ably expressed through the Local Centres now established. This activity has not confined itself to Local Centres only, but has extended into a wide field of professional organisation with most valuable results.

### The College Register.

The College can now be justly recognised as the central organising body, representing the trained nurses of this country, registering as it does at the close of its financial year 17,336 general-trained nurses. A Register of Nurses of such strength has never before been compiled in this country, and it is already receiving the recognition due to it. The Council, realising the importance of the Register, felt justified in incurring the expense of having it printed and published, and this great task was carried out in much detail and great accuracy. The Register has proved of valuable assistance to public and other bodies requiring reliable information as to the qualifications essential for a skilled nurse.

### State Registration for Nurses.

The College records with great satisfaction the passing of the Bill, introduced by the Minister of Health, for the registration of Nurses for the Sick in England and Wales, together with similar Bills for Scotland and Ireland, thus completing the programme of State recognition for all classes of nurses. The Bills received the Royal Assent, and thus became Acts of Parliament on December 23, 1919. The thanks of the profession are due to the Minister, Dr. Addison, for securing the adoption of a measure which embodies the principle for which the College has always stood and fought—that a substantial majority of the General Nursing Council set up under the Act should be elected by the nurses on the General Register.

The Council, having been invited by the Minister of Health to submit names for appointment on the General Nursing Council to be constituted under the Nurses Registration Act for England and Wales,* sent, on January 16, 1920, the following list, as the result of a postal ballot of Council Members:—

Miss Barton, R.R.C.; Dame Sidney Browne, G.B.E., R.R.C.; Miss Clark, R.R.C.; Miss Cox-Davies, R.R.C.; Miss Gibson; Miss Hogg, C.B.E.; Miss McIntosh, C.B.E., R.R.C.; Miss Monk, R.R.C.; Miss Musson, R.R.C.; Miss Peterkin; Miss Reed; Miss Sparshott, C.B.E., R.R.C.; Miss Lloyd Still, C.B.E., R.R.C.

To celebrate the passing of the Act the Chairman and the Council were "At Home" at the Royal Automobile Club on January 15, 1920, and among the guests were the Minister of Health, with the late Sir Robert Morant, Viscount Sandhurst—who was responsible for the Bill in the House of Lords—and over 400 nurses and their friends.

### Disabled Nurses.

In September last the Minister of Labour was approached with a view to obtaining grants for the training of disabled nurses in other occupations, and approved a scheme, which was soon after put into operation, and from which many of our members have already benefited.

### Salaries Committee.

After the circulation of the Report of the Special Committee on Salaries and Conditions of Employment of Nurses the Council is able to report considerable co-operation and assistance from hospitals and other institutions in endeavouring to afford better conditions and salaries for trained nurses. It has also been possible to improve many salaries through the Appointments Bureau of the College, which has often been able to induce employers to pay higher salaries than they originally offered to applicants for vacancies. Through the intervention of the College members of the Royal Naval Nursing Services are granted gratuities not hitherto allowed, and a letter has recently been addressed to the Director-General drawing attention to the excessive hours of duty in the Naval Service. A reply has been received that an inquiry is being held on the matter. Although there is satisfaction in knowing that the conditions of service in the profession have improved, the Council will not cease its efforts in this direction.

### Hours of Employment Bill.

The Council has to record its support of the Hours of Employment Bill contingent upon the introduction of modifications suited to the particular needs of the profession. In this connection it approached the Ministry of Labour by correspondence, and also through a deputation which was received by the Minister's Permanent Secretary, Sir David Shackleton.

The Council has appointed a Committee to consider all legislative measures which are thought to affect the trained nurse, such as the Unemployment Insurance Bill and the Hours of Employment Bill.

* The names of the College members selected by the Minister were published in our issue of May 1, p. 127.—Ed. The Hospital.

### Local Centres.

The number of Local Centres now established is twenty-three, and the Council cannot thank sufficiently the members of those Centres for the active support they have given through the past year, especially with reference to the passing of the Nurses Registration Act and the augmentation of the Nation's Fund for Nurses, and by showing a keen desire to co-operate with the Council in the various undertakings it has in hand. There are now in connection with the Centres, in addition to Clubrooms, four Residential Clubs, and many others are anticipated. The members are showing their aptitude for organisation by the practical manner in which they are helping to build up the Centres and bringing before their colleagues and the public professional matters of importance. They are greatly to be commended in supporting the educational policy of the College by the establishment of Local Scholarships and their contributions to the proposed Chair of Nursing.

### Organising Secretary and the League of Red Cross Societies.

The Council congratulates Miss Gertrude Cowlin on her appointment as Assistant Director of the League of Red Cross Societies at Geneva, and desires to express its appreciation of the valuable pioneer work she did for the College in establishing the Local Centres throughout the country, which will remain as a lasting memorial of her energy, tact, and enthusiasm.

### The College Bulletin.

The Council announces with much pleasure the first issue of the College Bulletin, which it is proposed to circulate quarterly, the first year free of charge to members. It is hoped that it will be the means of keeping each member in closer touch with the work of the College and of cementing that spirit of unity which has previously prevailed. The Council, however, realises that it must continue to rely on the Nursing and General Press to bring the activities of the College before the Profession at large and before the Public, and it takes this occasion of again expressing its appreciation of the sympathetic and valuable support thus extended since the establishment of the College.

### The Badge.

Another factor in the development of *esprit de corps* amongst members will be found in the issue of the Badge, which is widely approved.

### The British Women's Hospital Committee and Nation's Fund for Nurses.

The Council would again bring to the notice of members the gratitude due to the British Women's Hospital Committee for its generous help towards the Funds of the College and for the establishment of a Tribute Fund for nurses broken in health. The Annual Balance Sheet testifies to the success of the efforts of this Committee. The British Women's Hospital Committee, having raised the sum it set out to do, has now transferred the Nation's Fund to a Council, including the Members of the College Council, a few members of the British Women's Hospital Committee, and other persons particularly interested in the Profession. The Council of the College feels it may continue to rely on its members to do all in their power to augment the funds of the College, as well as to meet, as far as possible, the needs of the sick.

The Council also records with gratification the generosity of Viscount Burnham, in opening the columns of the *Daily Telegraph* for a "Nurses Shilling Fund," affording an opportunity for the support of the small contributor.

The Tribute Fund has been able to enlarge its scope and has been the means of assisting many nurses broken in health, whether members of the College or not, who otherwise would have had to seek refuge in Poor Law Infirmaries.

### Studentships.

The Council is happy in being able to report that Studentships have again been awarded for the purpose of qualifying members as Sister-Tutors. Thirty-seven candidates presented themselves for the examination, thirty-one of whom sat in London, three in Scotland, and three in Ireland. The Selection Committee in awarding the Studentships again paid special attention to the past experience and references of the candidates, and the total number of marks gained in the qualifying examination was only one factor in the selection of the successful competitor.

Eight Studentships were awarded, three by the Council of the College, three by an anonymous donor, and two by Lady Cowdray. The successful students were as follows: Miss D. M. Davis, Miss E. M. Edmunds, Miss A. M. Rose, Miss F. M. F. Bowdler, Miss N. H. MacCheane, Miss M. L. Lane, Miss D. Windley, and Miss Hitch.

These eight students took up their residence at King's College for Women in October last, and will conclude their studies in June.

The College is indebted to Lady Cowdray for the endowment of two Studentships named the "Cowdray Studentships," to enable Trained Nurses to take a course of study extending over one year, to fit them for the appointment of Sister Tutor in a Nurse Training School. Candidates for the Cowdray Studentships must be members of the College, who have received their professional training either in Aberdeenshire or Sussex.

### Supervision of Nursing Studies.

Having in view the increasing number of nurses who may go to King's College for Women, Campden Hill, the Council realises the importance eventually of making provision for their studies to be supervised by a trained nurse. A scheme is, therefore, under consideration to carry out such a suggestion.

It is hoped that the time may not be so far distant when a Chair of Nursing may be established at one of our Universities, and an Endowment Fund has been initiated for this purpose.

Midwifery Grants.

To assist members to obtain the Certificate of the Central Midwives Board the Council last December awarded grants of £15 each to fifteen members. There is every evidence that this financial assistance is much valued by the recipients, the result of which, it is hoped, will be an increased number of trained nurses practising midwifery.

Appointments and Enquiry Bureau.

This department, opened the week of the signing of the Armistice, and organised to meet the needs of members seeking resettlement into civil life, has fulfilled its purpose as regards appointments, of which over a hundred have been made, including some very good posts abroad as well as at home. It is not proposed to continue this branch of the College's activities, except the Enquiry Department, which will now be converted into an Information and Enquiry Bureau, and which, it is anticipated, will be of valuable assistance to members and others seeking information on matters concerning nursing and public health.

Legal Advice.

A great many members have availed themselves of the legal advice afforded them, and in this connection the Council would place on record the indebtedness of the College to Sir Charles Russell, who generously refuses any fees for what has proved to be a great demand on his time and attention. How much his courtesy and sympathetic interest have been appreciated, many of our members can testify.

Loan Fund.

The Council has decided to set aside at an early date in its new financial year a fund "from which loans may be made to members of the College needing assistance to qualify them in any particular branch of nursing or for other purposes." The necessary forms and documents have now been passed by the Council, and a Committee has been appointed for the purpose of carrying out the work.

Conferences.

During the year the Council has received invitations from the following, amongst other, societies to send representatives to conferences:—The National Society for Prevention of Tuberculosis; the National Council of Women, with reference to the League of Nations; the Royal Sanitary Institute; the Women's Industrial League; the Professional Classes War Relief Society; the Council of Professional Women; the British Federation of Medical and Allied Societies.

The Council is represented on:—The Watching Council, the Ministry of Reconstruction; the Nurses' Resettlement Committee, Ministry of Labour.

Affiliations.

The College is affiliated to the British Federation of Medical and Allied Societies: Representatives:—Dame Sidney Browne, G.B.E., R.R.C., Mrs. Cecil Carter, A.R.R.C., Comyns Berkeley, Esq., M.C. (Cantab.). The National Council of Women: Representatives:—Public Health and Insurance Sectional Committee, Miss Henry; Parliamentary and Legislation Sectional Committee, Miss Cox-Davies; Mothercraft and Child Welfare Exhibitions Committee, Miss Peterkin; Education Sectional Committee, Miss Lloyd Still.

Ministry of Health, Consultative Council.

It is a matter of great regret that no representative of the Nursing Profession has been appointed to sit on the Consultative Council under the Ministry of Health, in spite of the fact that the College was asked to submit nominations. Although indirect means are provided for expert nursing opinion to be obtained through an Advisory Committee, the Council is strongly of opinion that in matters of health, in which the trained nurse plays such an important part, direct representation upon the Consultative Committee should have been at once conceded.

Nurse Training Schools Visited.

The Council has accepted invitations from the Committees of four Nurse Training Schools to send representatives to visit the Schools with a view to the recognition of the Certificate as a qualification for membership of the College. In three instances the school has accepted, and put into effect, the recommendations of the Council made on the Report of its representatives, and in the fourth instance the Council is waiting for the adoption of its recommendations before admitting the Certificate as a qualification for membership of the College.

Bonchurch Home.

The Council is able to report the purchase of a Cottage at Bonchurch, Isle-of-Wight, for a Home of Rest for nurses, made possible through the generosity of the British Women's Hospital Committee and Mrs. Martin Harvey. The Home, which is beautifully situated, will shortly be opened, and it is anticipated there will be very beneficial results to members needing rest and change. A sum of £4,000 has also been received by the College from the British Farmers' Association towards the provision of Homes of Rest for Nurses.

Election of Council.

The election this year marks the retirement of the last remaining third of the first nominated Council, so that from now onwards the responsibility of selection of those who are to carry on the great work of the College rests entirely wtih the members.

Resignations and Deaths.

There have been during the year nine resignations, and the Council regrets to record the death of fifty-two members.

(Signed) ARTHUR STANLEY, *Chairman.*
M. S. RUNDLE, *Secretary.*

April 12, 1920.

### FIFTH ANNUAL REPORT OF THE HON. TREASURERS.

The Hon. Treasurers have pleasure in submitting the audited accounts of the College for the year from April 1, 1919, to March 31, 1920. During this period 4,322 members were registered and paid their fees, as against 6,047 in the previous year. The audited accounts of the Scottish and Irish Boards are also submitted. A grant of £400 to the Scottish Board and £415 to the Irish Board has been paid during the year.

The form in which the accounts are presented this year differs somewhat from that adopted in previous years. The alteration is caused by the receipt of several donations specially earmarked to specific funds. The effect of opening these separate funds is to reduce the amount of the donations towards the General Fund, and as a result there is a deficit on the year's working of £782 5s. 1d., but it must be borne in mind that there is nothing charged against the special funds to represent a fair proportion of the cost of administration. It will be observed that the accumulated balances on the funds amounted, at March 31, 1920, to £56,783 12s. 1d. Included in the expenses of management are those of printing the Register, which is of necessity a very costly procedure. The expenditure on stamps has been exceptionally heavy, 75,626 communications having been posted from the head office during the year. Such extensive correspondence, together with the work of keeping the Register up to date, demands more labour each year, and entails the employment of an increased clerical staff.

The Council, having decided to assist financially College members who wish to train for the Certificate of the Central Midwives Board, has set aside for this purpose £225, of which £90 has already been spent, enabling six members to receive £15 each towards the expense of this training. £365 8s. 0d. has also been allotted to studentships by the College, and in addition the Council received from an anonymous donor £256 11s. 0d. for three studentships, and from Lady Cowdray £172 18s. 0d. for two other studentships. The expenses incurred by the Salaries Committee is money well spent, the recommendations of the Committee resulting, as they have, in a reorganisation of the hours of duty and in an increase in the salaries of nurses in a large number of public institutions.

All members are to be congratulated on the possession of a "Nurses' Rest Home Fund," amounting to £3,728 2s. 9d., and the Irish members, owing to the generosity of Lord Iveagh, on the investment of £1,000 for the Dublin Nurses' Club.

(Signed) COMYNS BERKELEY,
SIDNEY BROWNE,
W. H. GOSCHEN,
*Hon. Treasurers.*

### SCOTTISH BOARD OF THE COLLEGE, 8 Drumsheugh Gardens, Edinburgh.

ANNUAL REPORT, 1919-20.

Since the last Annual Report was issued the Scottish Board has met four times, the General Purposes Committee five times, and the Registration Committee ten times.

The first Scottish Board nominated by the Council of the College ceased to exist after July last, and the new Board was formed which, in accordance with the Constitution, consisted of thirty members, fifteen of these being elected by postal ballot by members of the College on the Scottish Register. Professor Ritchie, of Edinburgh University, who was elected Chairman on the formation of the first Board, regretted that he was no longer able to hold this office. Professor Glaister, of Glasgow University, was elected in his place, Dr. Ker (the representative of the Royal College of Physicians, Edinburgh, on the Scottish Board), Vice-Chairman, and Miss Gill, R.R.C., Hon. Secretary and Treasurer.

The Board desires to express its thanks and appreciation of the very successful work done by Professor Ritchie, during his period of office, to Miss Gill as Hon. Secretary and Treasurer, and to Colonel Mackintosh, Chairman of the Registration Committee, to which office he has been re-elected.

During the year much thought has been given and work done to promote the State Registration of Nurses, and in this connection the Board expresses its thanks to Scottish members of the College Council for the active part they took in this in watching in London the interests of the members of the College in Scotland.

The membership of the College in Scotland has made a distinct advance, the total number of members trained in Scotland being now 1,869.

The returns for the year show an increase of 94 in the number who joined during the year, as compared with the number who joined in 1918-19. During the year the Registration Committee has considered 591 applications, 535 of which have been recommended to the Council for admission to the Register, fourteen are still under consideration, eighteen were found to be ineligible, sixteen were cancelled, seven withdrawn, and one applicant is since deceased. The applications of seventy-five further candidates remain to be dealt with.

The interest in the College in Scotland has been very marked during the year, and the work of the Local Centres has proved their value as agents in the advancement of the objects of the College.

The Board acknowledges with much appreciation the work done by the Local Centres in connection with the Bills for the State Registration of Nurses which were promoted before the Government Bills, entailing as it did a great amount of clerical work on the part of the members. The Board also records with pleasure that the Club so long wished for by the Edinburgh members is now established.

In Glasgow the membership has increased, and there also an active policy is maintained, lectures and social gatherings being organised, and interest in the College is thus stimulated.

Two new centres have been formed during the year: (1) Aberdeen; (2) Dundee. The Aberdeen Centre hopes soon to have a club for its members. Dundee has also made good progress in the short time it has been formed, and in connection with this Centre a Rest Home at Carnoustie, for members in Dundee and district, is in course of being established.

Both centres have an educational policy, and have regard to the social side in order to bring the members in touch with each other, and to be of real benefit to them.

The State Registration of Nurses is now an accomplished fact, Acts of Parliament for England and Wales, for Scotland, and for Ireland having been passed.

The Board was invited by the Scottish Board of Health to submit the names of persons whom they would recommend, if appointed, to serve on the Consultative Council for the Medical and Allied Services, under the Ministry of Health, and also on the General Nursing Council for Scotland under the Nurses Registration (Scotland) Act.

The Board feels that the College and all it stands for is thus being distinctly recognised as a body of nurses which must be considered in questions of importance affecting the profession, and learns with pleasure that Miss Gill, R.R.C., has been appointed to the former Consultative Council.

The Board is glad to report that the aims of the College are becoming more widely known in Scotland, and much real progress has been made.

JOHN GLAISTER, *Chairman.*

C. A. PIKE, *Secretary.*

### IRISH BOARD OF THE COLLEGE, 54 Fitzwilliam Square, Dublin.

ANNUAL REPORT, 1919-20.

The College year now concluded has been one of distinct progress for the Irish Branch, and, though many difficulties have had to be encountered and every step of the way fought, the position of the College in Ireland to-day is very much stronger than it was a year ago.

At the date of the last report the number of Irish-trained members on the College Register was 521. Since then 319 have paid their fees, making a total of 840. There are 9 Irish-trained nurses whose applications have been accepted but who have not yet paid their fees, while many are still under consideration. There are also 322 members trained in England or Scotland but working or having their homes in Ireland.

When the Minister of Health first made it known that the nurses of Ireland would not come within his jurisdiction in the matter of State Registration the Irish Board took an active part in securing an Act for the Registration of Irish Nurses similar to the English Act, and on the General Nursing Council for Ireland appointed under this Act there are four College members, three of whom were appointed as representatives of the College in Ireland. Every effort will be made by the College, through its representatives, to ensure that in its administration the Act will fulfil all expectations and do much to raise the educational and professional standards of nursing in the future.

In its last report the Board noted a sudden increase of public interest in the economic conditions of the nursing profession. Many reforms in the salaries and hours of hospital nurses have taken place since then, and the influence of the College has been strong in upholding the necessity for progress in this direction. The Irish Board has been the direct means of securing better conditions for some of its members working in Union infirmaries, and is now considering steps to be taken in conjunction with other nursing organisations to secure a general improvement by regulation of the salaries of Poor-Law Nurses throughout the country, thus protecting the nurses and obviating the necessity for frequent and protracted negotiations with individual Boards of Guardians.

The Irish Branch, working on the same democratic principle as the College Council, is anxious that its members should avail themselves of the opportunities of the postal ballot and seek fuller representation on the Board. At the last election five entirely new members were elected, four of whom were nurses not in the position of Matron. This is an encouraging proof of increasing alertness in the profession, and it is expected that this year there will be still greater interest in what may be called the local elections.

As regards the educational aspect of the work of the College, the Irish Board is very glad to note that the idea of providing Sister-Tutors for the teaching work in hospitals has been taken up by several Dublin hospitals. It is hoped that this will greatly increase the interest felt by Irish College members in the Sister-Tutor Studentships offered by the College, and that there will be no lack of suitable Irish candidates.

A great advance in meeting the social needs of Irish nurses has been made during the past year. In August the Board moved its offices from 23 Kildare Street, and now has its headquarters in Fitzwilliam Square, one of the most pleasant residential quarters in the city. A whole house has been rented, and that part not required by the College has been established as a Residential Club for members of the nursing profession. This Club not only provides bedroom accommodation for nurses passing through Dublin, but also caters for non-residential members, who have the free use of the Clubrooms, can obtain any of their meals there, can entertain their friends, and avail themselves of the lending library. It thus not only meets an urgent need, but also acts as a Local Centre of the College in bringing nurses together and making possible an untrammelled interchange of thought and ideas, and a freer social intercourse than is usually open to nurses.

Nurses are availing themselves more and more freely of the Irish headquarters as a bureau of information, and are beginning to recognise that the College is genuinely anxious to help them and always ready to take any trouble on their behalf. A feeling of confidence is thus being created which will do much to counteract the proverbial apathy of nurses, and will help to inspire them with courage and hopefulness in tackling their many problems and difficulties. When nurses learn the true meaning of the word "co-operation," then the necessary foundations will be complete, and a rapid advance will be practicable. The Irish Board are thus able to look forward confidently to sure and steady progress towards the attainment in this country of the splendid objects of the College.

(Signed) GEORGE PEACOCKE, *Chairman.*

MARY J. CHISHOLM, *Hon. Secretary.*

*April* 1920.

MR. ROBERT T. LAWLOR has resigned the position of Secretary to the London Temperance Hospital, having accepted the secretaryship of the St. Paul's Hospital. Mr. Lawlor has been for sixteen years in the service of hospitals in London—nearly thirteen years at the West End Hospital for Nervous Diseases, and since January 1917 Secretary of the London Temperance Hospital.

